# Altered resting-state brain activity in functional dyspepsia patients: a coordinate-based meta-analysis

**DOI:** 10.3389/fnins.2023.1174287

**Published:** 2023-05-12

**Authors:** Yangke Mao, Pan Zhang, Ruirui Sun, Xinyue Zhang, Yuqi He, Siyang Li, Tao Yin, Fang Zeng

**Affiliations:** ^1^Acupuncture and Tuina School, Chengdu University of Traditional Chinese Medicine, Chengdu, China; ^2^Acupuncture and Brain Science Research Center, Chengdu University of Traditional Chinese Medicine, Chengdu, China; ^3^Key Laboratory of Sichuan Province for Acupuncture and Chronobiology, Chengdu, China

**Keywords:** functional dyspepsia, neuroimaging, insula, fMRI, meta-analysis

## Abstract

**Background:**

Neuroimaging studies have identified aberrant activity patterns in multiple brain regions in functional dyspepsia (FD) patients. However, due to the differences in study design, these previous findings are inconsistent, and the underlying neuropathological characteristics of FD remain unclear.

**Methods:**

Eight databases were systematically searched for literature from inception to October 2022 with the keywords “Functional dyspepsia” and “Neuroimaging.” Thereafter, the anisotropic effect size signed the differential mapping (AES-SDM) approach that was applied to meta-analyze the aberrant brain activity pattern of FD patients.

**Results:**

A total of 11 articles with 260 FD patients and 202 healthy controls (HCs) were included. The AES-SDM meta-analysis demonstrated that FD patients manifested increased activity in the bilateral insula, left anterior cingulate gyrus, bilateral thalamus, right precentral gyrus, left supplementary motor area, right putamen, and left rectus gyrus and decreased functional activity in the right cerebellum compared to the HCs. Sensitivity analysis showed that all these above regions were highly reproducible, and no significant publication bias was detected.

**Conclusion:**

The current study demonstrated that FD patients had significantly abnormal activity patterns in several brain regions involved in visceral sensation perception, pain modulation, and emotion regulation, which provided an integrated insight into the neuropathological characteristics of FD.

## Introduction

Functional dyspepsia (FD), a prevalent functional gastrointestinal disorder (FGID), is characterized by a group of long-term and fluctuating symptoms including postprandial fullness, early satiety, epigastric pain, and epigastric burning without a clear structural explanation (Enck et al., 2017). As one of the most common FGIDs, FD has a high incidence, affecting up to 10–16% of the individuals in the general population worldwide (Ford et al., 2020). According to previous studies, the population prevalence of FD ranges from 8% to 23% in Asia and approximately 12% in North America (Ghoshal et al., 2011; Aziz et al., 2018). Although not life-threatening or disabling, FD imposes a heavy medical burden on individuals and significantly reduces their quality of life (El-Serag and Talley, 2003; Miwa et al., 2022). However, due to the incomplete understanding of its pathophysiology and lack of specific biomarkers, the diagnosis and treatment of FD are largely restricted.

There is increasing evidence indicating that FD is a complex disease with multi-factorial interaction (Tack et al., 2004). Central homeostasis imbalance (Tait and Sayuk, 2021), gastroduodenal motility alteration (Oustamanolakis and Tack, 2012), gastroduodenal hypersensitivity (Mertz et al., 1998), and intestinal microbiota disorder (Shimura et al., 2016) have been considered as the potential pathogenesis of FD. Specifically, in the latest Rome IV criteria (Drossman et al., 2018), FD is defined as a functional disorder of brain–gut interaction, which indicates that the dysfunction of the alimentary tract and brain, as well as aberrant interaction patterns between them, play an important role in explaining the mechanism of FD. Within this framework, researchers utilized real-time neuroimaging technology to explore how the brain activity patterns of FD patients differ from that of healthy controls (HCs) under the resting state and stimulus conditions. With the application of functional neuroimaging technology, multiple aberrant brain regions have been found in FD patients, including but not limited to the insula (Zeng et al., 2012), thalamus (Liu et al., 2018), amygdala (Zeng et al., 2019), and dorsolateral prefrontal cortex (Liu et al., 2017). These functional brain abnormalities were identified in a systematic review (Lee et al., 2016). However, the differences in sample size, scanning methods, imaging modalities, data analysis, and other methodological limitations may lead to the heterogeneity of results, making it difficult to draw reliable and comprehensive conclusions. Therefore, it is necessary to use a meta-analysis method to make a comprehensive analysis of the previous findings.

Anisotropic effect size signed differential mapping (AES-SDM) is a potent method for coordinate-based meta-analysis of neuroimaging data (Müller et al., 2018). It provides a statistical technique for pooling different neuroimaging findings and integrating heterogeneous brain regions based on the peak coordinates and statistical parametric maps (Radua and Mataix-Cols, 2009; Radua et al., 2012, 2014). Compared with another commonly used method, activation likelihood estimation (ALE), AES-SDM could test publication bias, reproducibility, and robustness of the results (Radua et al., 2012). With the advantages of high sensitivity, good control of false positives, and lower imprecision than other coordinate-based methods, AES-SDM has been widely used and proven in recent studies (Ma et al., 2022; Zhang et al., 2023).

Therefore, the current study aimed to utilize the AES-SDM method to identify the functional brain alterations of FD patients under resting-state conditions. The results of this study would provide a clearer understanding of the underlying pathophysiological mechanism of FD and contribute to finding potential neural biomarkers, thus facilitating the advancement of FD diagnosis and treatment.

## Materials and methods

This study was performed in accordance with the Preferred Reporting Items for Systematic Reviews and Meta-Analyses (PRISMA) guidelines (Page et al., 2021) and was registered at the International Prospective Register of Systematic Reviews of the University of York (PROSPERO, registration no. CRD42019134983).

### Search strategies

A comprehensive search was conducted from the following eight electronic databases from inception to 15 October 2022: PubMed, Web of Science, Embase, Cochrane Database, China National Knowledge Infrastructure, Chongqing VIP Database, China Biology Medicine Disc Database, and Wanfang Database. The language was restricted to English or Chinese. The search terms of PubMed were as follows: (“functional dyspepsia” OR “indigestion” OR “FD”) AND (“neuroimaging” OR “magnetic resonance imaging” OR “MRI” OR “positron emission tomography” OR “PET” OR “single photon emission computed tomography” OR “SPECT” OR “amplitude of low-frequency fluctuation” OR “ALFF” OR “fractional amplitude of low-frequency fluctuation” OR “fALFF” OR “regional homogeneity” OR “ReHo” OR “independent component analysis” OR “functional connectivity”). The search strategies of the other seven databases were modified according to the above formula. After the electronic searches, we further screened the references of relevant reviews and included articles to find potential studies.

### Inclusion and exclusion criteria

Studies were included if they met the following criteria: (1) all subjects were above 18 years and patients met the Rome criteria for FD; (2) those that had taken resting-state functional neuroimaging as the imaging technique; (3) those that focused on the difference of voxel-wise brain activity between FD patients comparing to HCs; (4) results obtained with whole-brain analysis and reported in the Talairach or Montreal Neurological Institute (MNI) coordinates; and (5) original article published in a peer-viewed journal.

Studies were excluded if they had the following features: (1) reviews, protocols, conference articles, letters, animal studies, or case reports; (2) patients underwent any interventions or stimuli during or prior to neuroimaging scan; and (3) the enrolled FD patients were combined with other diseases.

### Data extraction

Two authors independently screened the identified articles and then reviewed the full text to check whether the articles met the inclusion and exclusion criteria. Any disagreements or uncertainties were discussed in the consultation and resolved by the third author. The following study information was acquired: (1) basic information (name of the first author and publication year), (2) methodology (sample size of patients and HCs, diagnostic criteria of patients, and details of neuroimaging method including scanning device and imaging modality), and (3) difference in functional brain activity between FD patients and HCs (peak coordinates of different regions in Talairach or MNI space, cluster size, and statistical threshold). If the included study reported the altered brain regions without specific coordinates, we would contact the authors to request detailed information.

### Quality assessment

A 12-point checklist was adopted to assess the quality of all included studies according to previous neuroimaging meta-analyses (Du et al., 2014; Zhang et al., 2023) (Supplementary Table S1). The customized checklist focused on clinical and demographic information, neuroimaging methodology, and the quality of results. Quality assessment was conducted by two authors, respectively. The third author took the ultimate decision in the event of a disagreement.

### Statistical analysis

The coordinate-based mate-analysis study was conducted with AES-SDM software version 5.15 (https://www.sdmproject.com/software). The detailed procedure was summarized as follows. First, the peak coordinates and effect sizes (*T*-values) for the clusters of difference between FD and HCs were extracted from the published results. Then, the effect size and variance maps of brain function differences with an anisotropic Gaussian kernel were recreated (Radua et al., 2014). In order to balance sensitivity and specificity, the full width at half maximum of the Gaussian kernel was set to 20 mm (Radua et al., 2012). Subsequently, the standard meta-analysis was carried out to generate a mean map through voxel-wise calculation of the random-effects mean of the study maps, taking sample size, intra-study variability, and inter-study heterogeneity into account (Tang et al., 2018). Thresholds were set at a *P*-value of < 0.005 (voxel level), with peak height threshold *Z* > 1 and cluster size threshold ≥20 voxels (Radua et al., 2010). Results were presented on the standardized template in MNI coordinates.

To test the reproducibility and robustness of the results, a jackknife sensitivity analysis was conducted. If a cluster remained significant in all or most of the sensitivity analysis, this brain region was thought to be highly replicable (Radua and Mataix-Cols, 2009). For publication bias, the Egger test was used to evaluate the asymmetry of funnel plots. Any result with a *P*-value of < 0.05 indicated a statistically significant publication bias.

## Results

### Characteristics of included studies

**Figure 1:**
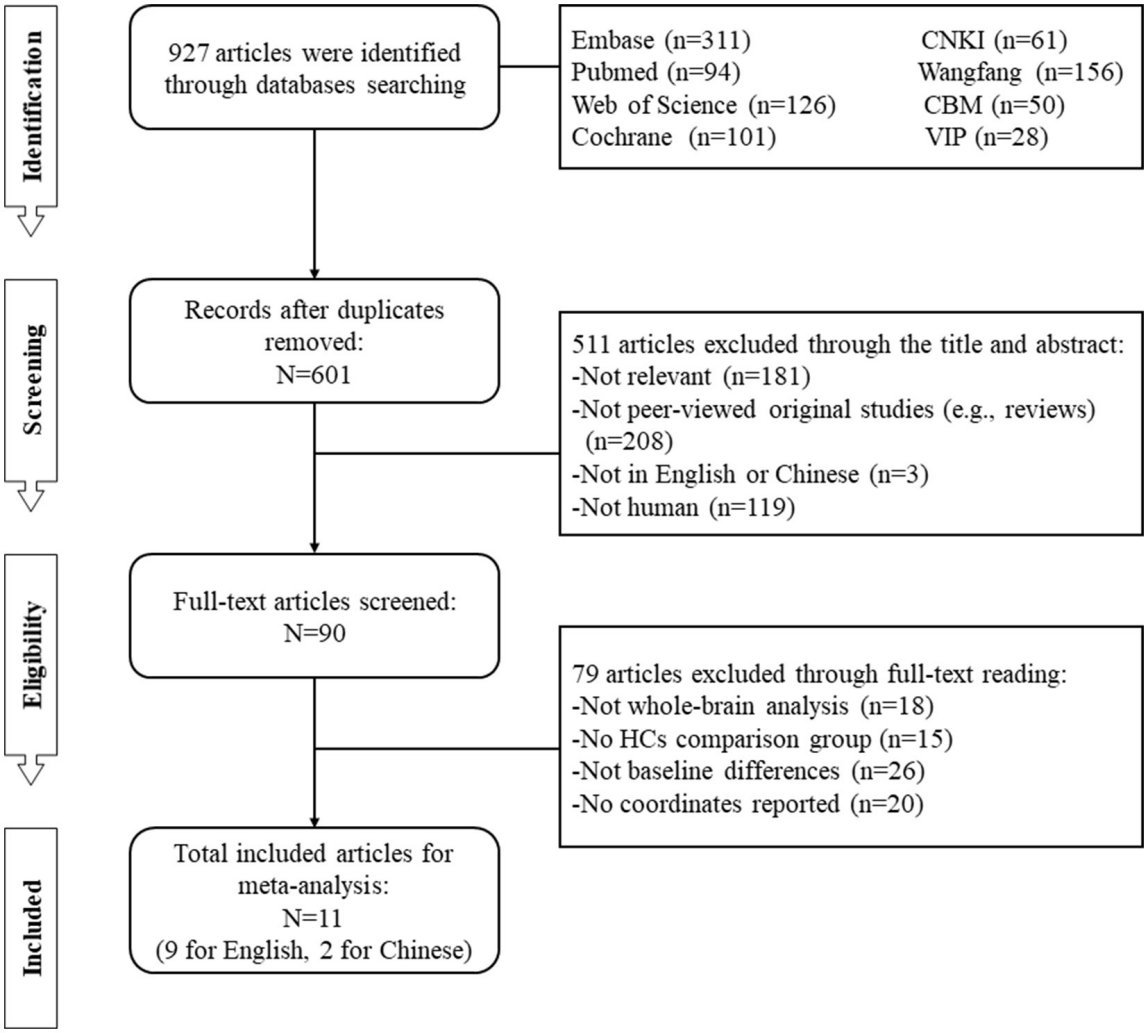
The procedure of retrieval and inclusion of studies.

A total of 927 articles were retrieved based on our search strategy, and 11 articles were finally qualified. The flowchart shows the procedure of retrieval and inclusion of studies (Figure 1). Since one of the studies included two subtypes of FD and performed between-group comparisons to HCs separately, it was split into two studies, as recommended in the previous study (Zhang et al., 2023). As a result, a total of 12 results were included in the meta-analysis to quantitatively investigate the brain abnormalities between FD patients and HCs. These studies included 260 FD patients (113 male subjects and 147 female subjects) and 202 HCs (84 male subjects and 118 female subjects). The detailed information on the included studies is displayed in Table 1.

**Table 1:** Detailed information of included studies.

**References**	**Sample size (female)**		**Mean age (SD)**		**Duration (SD) (month)**	**Imaging technique**	**Software**	**Analysis**	**Statistical threshold**	**QS (total 12)**
	**Patients**	**HCs**	**Patients**	**HCs**						
Van Oudenhove et al. (2010a)	25(20)	11(6)	33 ± 10	23.2 ± 1.7	NA	PET	SPM2.0	NA	*P*_FWE_ < 0.05	10.5
Zeng et al. (2009)	8(4)	8(4)	24.25 ± 2.49	25.50 ± 1.85	15.25 ± 4.20	PET-CT	SPM2.0	NA	*P*_Uncorrected_ < 0.005	10.5
Zeng et al. (2011)	39(20)	20(10)	22.87 ± 2.13	23.10 ± 1.86	22.74	PET-CT	SPM5	NA	*P*_FWE_ < 0.001	11
Liu et al. (2013)	30(20)	30(19)	22.5 ± 1.46	22.23 ± 0.94	35.77 ± 22.44	fMRI	SPM5	ReHo	*P*_Uncorrected_ < 0.001	11
Zhou et al. (2013)	29(10)	16(9)	22.41 ± 1.86	21.94 ± 0.85	33.72 ± 20.20	fMRI	FSL	fALFF	*P*_TFCE_ < 0.05	12
Ly et al. (2015)	12(11)	12(11)	29.8 ± 11.0	25.0 ± 13.7	NA	PET	SPM8	NA	*P*_FWE_ < 0.05	10.5
Chen et al. (2018)	28(17)	10(5)	38.94	42.3	NA	fMRI	SPM8/ REST	ReHo	*P*_Alphasim_ < 0.05	11
Lee et al. (2018)	12(7)	14(9)	46.46 ± 5.64	45.79 ± 4.71	156 ± 57.24	fMRI	SPM8	ALFF	*P*_FWE_ < 0.05	11.5
Qi et al. (2020)	18(7)	22(13)	43.78 ± 14.70	41.41 ± 13.04	48.94 ± 103.78	fMRI	SPM12	ALFF	*P*_GRF_ < 0.05	11.5
Qi et al. (2020)	13(6)	22(13)	41.77 ± 9.45	41.41 ± 13.04	53.96 ± 78.28	fMRI	SPM12	ALFF	*P*_GRF_ < 0.05	11.5
Guan et al. (2019b)	31(19)	19(11)	23.10 ± 2.49	22.89 ± 2.28	NA	fMRI	REST2.0	ReHo	*P*_Alphasim_ < 0.05	10.5
Guan et al. (2019a)	15(6)	18(8)	23.53 ± 2.90	22.67 ± 2.61	NA	fMRI	SPM12/REST2.0	ALFF	*P*_Alphasim_ < 0.05	10.5

ALFF, amplitude of low-frequency fluctuation; fALFF, fractional amplitude of low-frequency fluctuation; FSL, the functional MRI of the brain software library; fMRI, functional magnetic resonance imaging; FWE, family-wise error; GRF, Gaussian random field; HCs, healthy subjects; NA, not applicable; PET, positron emission computed tomography; PET-CT, positron emission computed tomography-computed tomography; QS, quality scores; REST, the resting-state fMRI data analysis toolkit; ReHo, regional homogeneity; SD, standard deviation; SPM, statistical parametric mapping; TFCE, threshold-free cluster enhancement.

### Abnormal functional brain activity in FD patients

**Figure 2:**
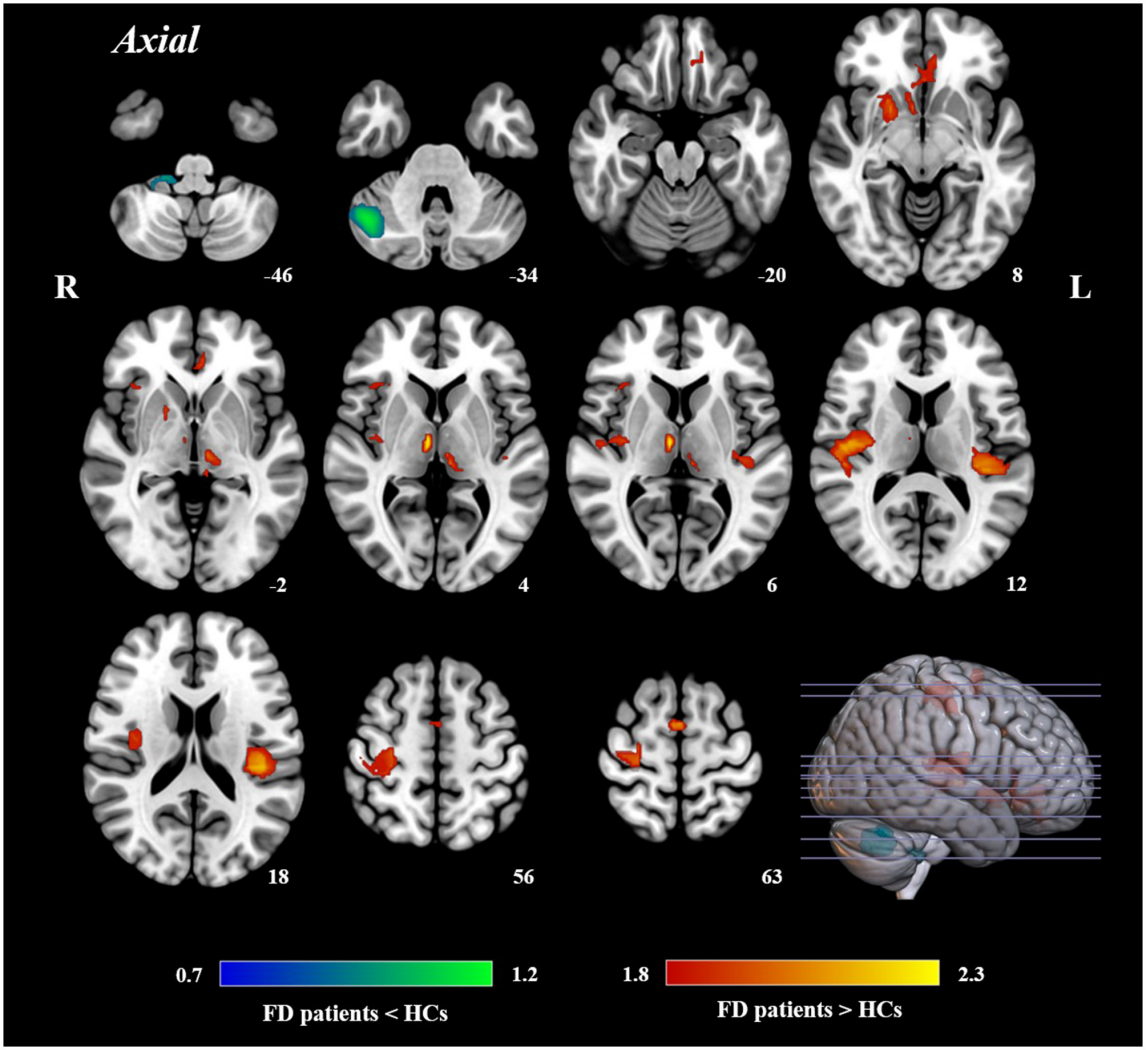
Results of AES-SDM of brain regions showing significant differences between FD patients and HCs.

The AES-SDM meta-analysis yielded a total of 12 clusters (Figure 2, Table 2). Compared to HCs, FD patients showed increased brain activity in the left insula/rolandic operculum, right insula, right precentral gyrus, left anterior cingulate/paracingulate gyrus (ACG), left supplementary motor area (SMA), right putamen, bilateral thalamus, and left rectus gyrus while decreased brain activity in the right crus I and the hemispheric lobule X of the right cerebellum.

**Table 2:** Brain regions showing significant brain activity differences between FD patients and HCs.

**Regions**	**Peak coordinates (MNI)**			***P*-value^a^**	**Z-score^b^**	**Voxels^c^**	**Cluster breakdown**
	**x**	**y**	**z**				
**FD>HCs**							
L insula/Rolandic operculum	−40	−24	20	0.0005	2.218	489	Left rolandic operculum, BA 48 Left insula, BA 48 Left superior temporal gyrus, BA 48 Left heschl gyrus, BA 48 Corpus callosum Left superior temporal gyrus, BA 41
R insula	38	−12	6	0.0018	1.993	423	Right insula, BA 48 Right rolandic operculum, BA 48 Right heschl gyrus, BA 48 Right superior temporal gyrus, BA 48
R precentral gyrus	26	−16	64	0.0013	2.042	359	Right precentral gyrus, BA 6 Right precentral gyrus, BA 4 Right superior longitudinal fasciculus II Right postcentral gyrus, BA 3 Right postcentral gyrus, BA 4
L anterior cingulate/paracingulate gyri	−6	40	−2	0.0015	2.023	200	Left anterior cingulate/paracingulate gyri, BA 11 Right striatum Right superior frontal gyrus, medial orbital, BA 11
L supplementary motor area	0	0	62	0.0009	2.105	114	Right supplementary motor area, BA 6 Left supplementary motor area
R lenticular nucleus, putamen	28	16	−8	0.0032	1.915	103	Right striatum Right lenticular nucleus, putamen
L thalamus	−6	−20	4	0.0009	2.122	100	Left anterior thalamic projections Left thalamus Corpus callosum
R thalamus	6	−14	6	0.0002	2.326	76	Right thalamus Right anterior thalamic projections
R insula	34	24	6	0.0012	2.058	38	Right insula
L rectus gyrus	−10	38	−20	0.0015	2.025	35	Corpus callosum
**FD**<**HCs**							
R cerebellum, crus I	44	−60	−34	0.0011	−1.171	489	Right cerebellum, crus I Right cerebellum, crus I, BA 37 Right cerebellum, crus II
R cerebellum, hemispheric lobule X	22	−36	−46	0.0015	−1.116	61	(undefined)

BA, Brodmann area; FD, functional dyspepsia; HCs, healthy subjects; L, left; MNI, Montreal Neurological Institute; R, right.

a Voxel probability threshold: *P* < 0.005.

b Peak height threshold: Z > 1.

c Cluster extent threshold: regions with < 20 voxels are not reported.

**Figure 3:**
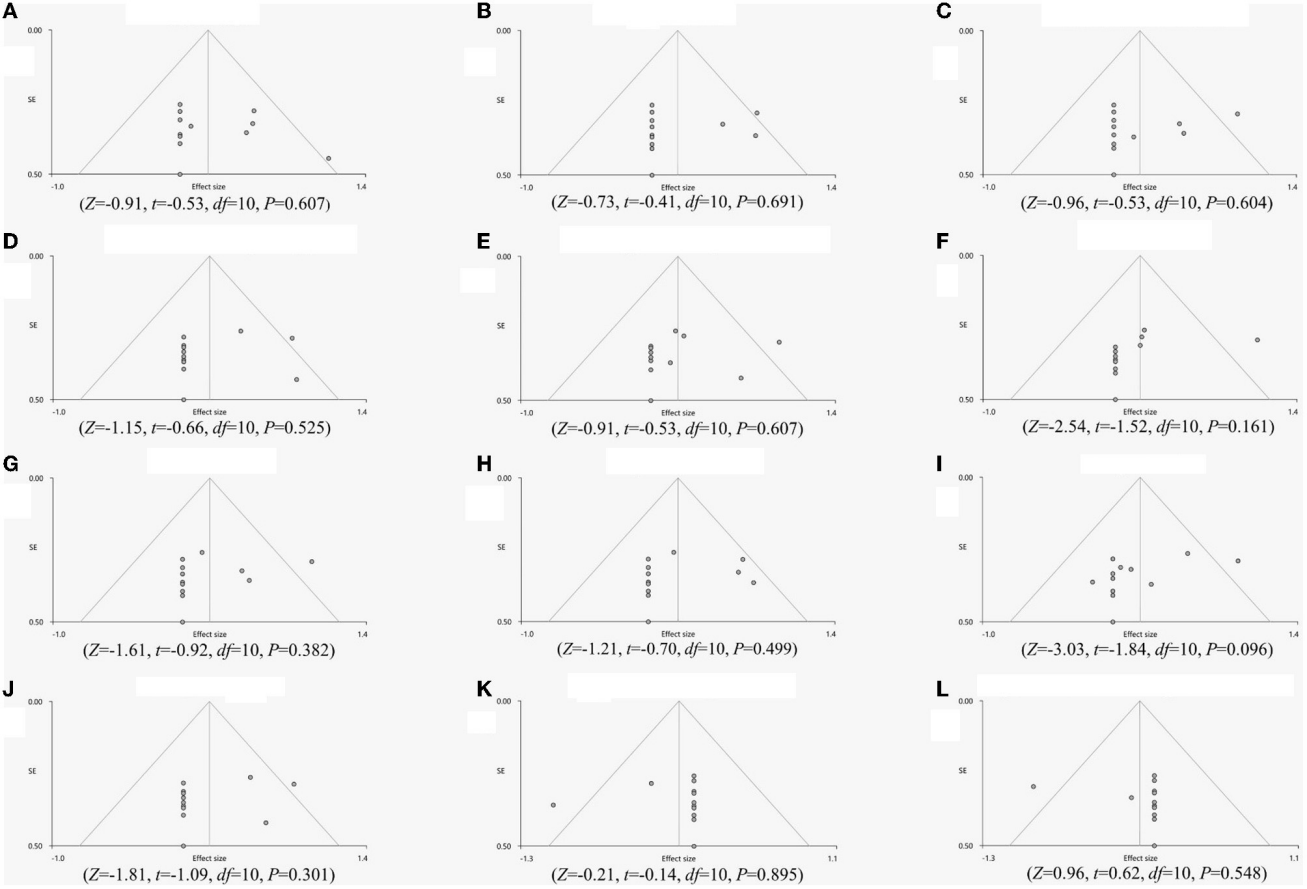
Egger's test of funnel plot asymmetry. **(A)** Left insula, **(B)** Right insula, **(C)** Right precentral gyrus, **(D)** Left anterior cingulate gyrus, **(E)** Left supplementary motor area, **(F)** Right putamen, **(G)** Left thalamus, **(H)**, Right thalamus, **(I)**, Right insula, **(J)**, Left rectus gyrus, **(K)** Right cerebellum, curs I, **(L)** Right cerebellum, hemispheric lobule X.

The whole-brain jackknife sensitivity analysis revealed that all regions were highly reproducible (Table 3). Egger's test of funnel plot asymmetry showed no significant publication bias of abnormal regions in FD patients compared to HCs (Figure 3).

**Table 3:** Sensitivity analysis of coordinate-based meta-analysis using the AES-SDM method.

**References**	**L INS**	**R INS**	**R PCG**	**L ACG**	**L SMA**	**R PUT**	**L THA**	**R THA**	**L RG**	**R CE (I)**	**R CE (X)**
Van Oudenhove et al. (2010a)	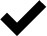	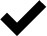	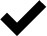	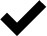	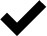	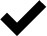	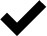	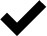	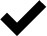	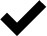	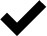
Zeng et al. (2009)	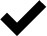	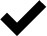	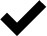	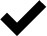	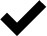	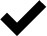	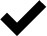	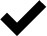	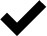	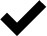	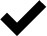
Zeng et al. (2011)	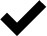	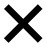	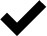	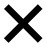	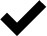	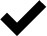	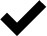	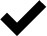	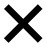	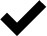	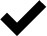
Liu et al. (2013)	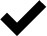	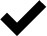	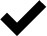	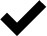	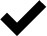	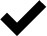	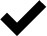	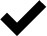	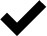	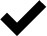	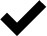
Zhou et al. (2013)	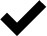	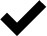	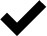	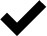	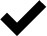	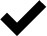	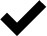	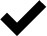	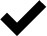	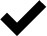	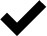
Ly et al. (2015)	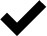	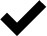	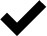	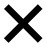	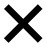	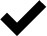	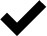	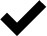	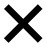	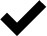	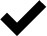
Chen et al. (2018)	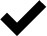	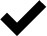	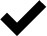	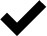	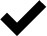	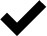	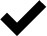	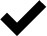	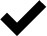	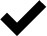	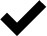
Lee et al. (2018)	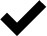	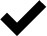	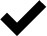	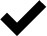	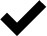	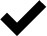	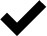	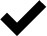	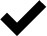	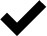	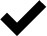
Qi et al. (2020)	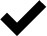	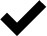	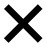	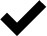	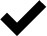	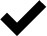	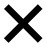	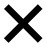	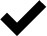	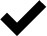	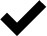
Qi et al. (2020)	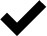	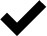	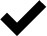	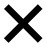	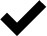	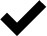	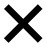	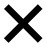	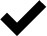	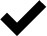	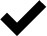
Guan et al. (2019b)	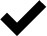	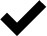	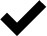	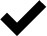	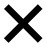	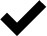	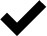	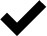	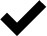	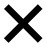	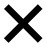
Guan et al. (2019a)	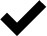	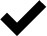	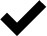	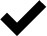	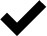	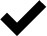	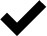	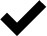	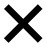	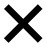	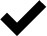
	12/12	11/12	10/12	10/12	10/12	12/12	10/12	10/12	9/12	10/12	11/12

ACG, anterior cingulate/paracingulate gyri; CE (I), cerebellum, (crus I); CE (X) cerebellum, (hemispheric lobule X); INS, insula; L, left. PCG, precentral gyrus; PUT, putamen; R, right; RG, rectus gyrus; SMA, supplementary motor area; THA, thalamus.

## Discussion

To the best of our knowledge, this was the first meta-analysis to investigate the alterations of brain activity in FD patients. The results showed with high reproducibility that FD patients had abnormal activity in several brain regions, including the bilateral insula, left ACG, bilateral thalamus, right precentral gyrus, left SMA, right putamen, left rectus gyrus, and right cerebellum.

Visceral sensation-related brain regions are thought to be key features of central pathological changes in FD. According to the results of the current study, FD patients showed significant brain abnormalities related to visceral sensation, especially in the insula, ACG, and thalamus. Among these areas, the alteration of the bilateral insula was the most notable finding, suggesting that the abnormal activity patterns of the insula may be one of the most important central pathological features of FD. The insula is a core region dealing with visceral sensation (Craig, 2002; Zubieta et al., 2005; Namkung et al., 2017). As shown in the previous studies, FD patients not only showed increased activity in the insula under resting-state conditions (Van Oudenhove et al., 2010b; Zeng et al., 2011) but also manifested abnormally higher activation during stimuli such as gastric distension (Van Oudenhove et al., 2010a). In addition, studies also found that aberrant functional connectivity strength in the insula correlated with disease duration (Liu et al., 2018), indicating that the chronic and recurrent symptoms of FD may exacerbate the abnormal visceral sensation. The cingulate cortex is considered to have a direct relationship with the internal neural of the gastrointestinal tract (Vogt, 2013). Thus, it is not unexpected that the increased activity of ACG was found in FD patients, which was in line with the previous functional magnetic resonance imaging and positron emission computed tomography studies (Zeng et al., 2011; Nan et al., 2015; Liu et al., 2018). For example, Nan et al. (2014) found that the regional homogeneity of the ACG was significantly strengthened and was positively associated with the severity of dyspeptic symptoms in FD patients. Furthermore, the altered brain activity in the thalamus, a subcortical region closely communicated with the ACG (Lee et al., 2016), was also detected in FD patients. As a crucial part of the subcortical network, the thalamus is a pivot for integrating and relaying sensations, especially visceral sensory signals (Sherman, 2007; Elvsåshagen et al., 2021), which plays a role in gastrointestinal sensory processing (Iannilli et al., 2014). It receives and primarily processes the afferent signals from the periphery and then relays them to higher-class brain areas such as the insula for sensory integration which correlates with visceral pain (Moisset et al., 2010; Mayer et al., 2015). In a recent resting-state study, FD patients had higher spontaneous activity in the homeostatic-afferent network which includes the thalamus and relates to visceral homeostatic-afferent information (Qi et al., 2020). Another task-state study observed robust bilateral thalamic activation during gastric distention (Ladabaum et al., 2001).

Interestingly, the insula, ACG, and thalamus are viewed as pivotal components of the “gastric sensation neuro-matrix” (Nagai et al., 2007; Van Oudenhove et al., 2008). Furthermore, the insula and ACG are also thought to be the essential part of the “pain matrix” (Jones, 1999; Naliboff et al., 2006), which play an important role in the pain process and regulation (Zubieta et al., 2005). They all receive gastrointestinal tract signals via the spinal/vagal pathway and are mainly involved in the processing of visceral sensations (Aziz et al., 2000). Given the close connection between the insula, ACG, thalamus, and visceral sensation, it is conceivable that the abnormal activity of the insula, ACG, and thalamus may be caused by the adverse sensation of chronic and recurrent gastroduodenal symptom attacks and thus further exacerbated the central dysregulation of dyspeptic symptoms.

There is also evidence proving that the putamen is combined with the thalamus in nociceptive information processing (Starr et al., 2011) and could contribute to the processing of sensory and motor aspects of pain (Bingel et al., 2004). The putamen was frequently activated during painful stimuli (Starr et al., 2011). In some chronic pain disorders, such as irritable bowel syndrome (IBS) and fibromyalgia, abnormal putamen was observed, suggesting that the putamen is strongly associated with the processing of pain-related motor responses (Song et al., 2006; Schmidt-Wilcke et al., 2007; Seminowicz et al., 2010). The cerebellum has been suggested to be involved in visceral activity as well as somatic balance and affective behavior (Allen et al., 1997). Many FD studies manifested abnormal activity patterns in the cerebellum. For example, Vandenberghe et al. (2007) found that FD patients showed activations in the bilateral cerebellum during painful gastric distension. Other FD studies also reported significant abnormalities of cerebellum activity in the resting state (Zhou et al., 2013; Chen et al., 2018). Similarly, some studies found similar altered cerebellar activations in patients with chronic visceral pain such as IBS (Guleria et al., 2017). These abnormal brain regions above implied that an altered pain network may be another crucial feature of FD. The abnormal brain activity above may represent an adverse response to nociceptive visceral projections, such as chronic pain and burning sensation from the upper abdomen. In addition, this inappropriate response possibly led to the dysregulation of pain sensation in the pain network.

Functional dyspepsia central alterations are characterized by disorders of multiple networks. In addition to visceral sensation and pain-related areas, pathological alterations involve emotion, cognition, etc. As reported in previous studies, FD patients always experience co-morbidity of gastrointestinal symptoms with emotional disturbances (Herrick et al., 2018). Liu et al. (2012) further found that FD patients with psychiatric disorders, especially anxiety and depression, had a higher glucose metabolism in the insula compared to FD patients without psychiatric disorders. Since the insula also mainly participated in emotion, homeostatic function, cognitive function, and affective function and not only visceral sensation (Cauda et al., 2011; Yu et al., 2020), it is plausible that the insula may largely be leading the process of visceral sensation and emotional modulation in FD patients.

Abnormal brain activity in the sensorimotor cortex, including the precentral gyrus and SMA, was found in this meta-analysis. The precentral gyrus, known as the primary motor area, as well as the SMA participate in movement control (Qi et al., 2020) and engage in somatic pain-related sensation (Morawetz et al., 2017; Li et al., 2021). Patients with somatic symptom disorder and pain symptoms demonstrated abnormalities in the precentral gyrus (Yoshino et al., 2014). Similarly, previous studies found activated SMA during visceral pain stimulation (Lotze et al., 2001; Kano et al., 2013). Therefore, these results suggested that the aberrant activity in the precentral gyrus and SMA may be attributed to the perception of abnormal gastrointestinal movements of FD patients.

As the current study utilized a relatively steady approach to identify the included studies, the results should be well representative of FD patients in the resting state. However, there were still several limitations. First, the limited number of resting-state neuroimaging studies of FD may introduce a risk of bias in the original results, which may require caution in interpreting the results. Second, the causality between FD and aberrant brain activity could not be verified. The alterations in brain regions are difficult to be elucidated because of one of the pathogenesis or the result of symptoms of FD due to the cross-sectional nature of the included studies. Third, language restrictions to English and Chinese might cause bias to some extent. Fourth, the voxel-wise meta-analysis was conducted according to peak coordinates and effect sizes rather than original brain images, which may affect the accuracy of the results. As a solution, the participant-level meta-analysis (Zunhammer et al., 2021) could be introduced in future studies.

## Conclusion

The current meta-analysis demonstrated multiple brain regions with abnormal functional activity in FD patients, including the insula, ACG, thalamus, precentral gyrus, SMA, putamen, and cerebellum. The abnormal brain regions could be interpreted as a consequence of the interaction between recurrent gastrointestinal symptoms and abnormal brain regulation. Importantly, these results manifested that the central pathological mechanism of FD represented as multi-network imbalance mainly involved visceral sensation perception as well as pain and emotion regulation. This study contributed positively to the interpretation of the central pathological features of FD, which would advance the understanding of its pathogenesis and may combine with machine learning, thus facilitating the diagnosis and treatment of the disease in the future (He et al., 2022).

## Data availability statement

The data analyzed in this study is subject to the following licenses/restrictions: The datasets analyzed in the current study are available from the corresponding authors upon reasonable request. Requests to access these datasets should be directed to FZ, zengfang@cdutcm.edu.cn.

## Author contributions

FZ and RS contributed to the conception and design of the study. PZ, XZ, YH, and SL retrieved studies and acquired data. YM conducted data analysis. YM and PZ wrote the article. TY and FZ revised the manuscript. All authors reviewed and approved the submitted version.
