# Supplementary material for: Altered resting-state brain activity in functional dyspepsia patients: a coordinate-based meta-analysis
Source: Front Neurosci. 2023 May 12;17:1174287. doi: 10.3389/fnins.2023.1174287 (PMC10213416; doi:10.3389/fnins.2023.1174287)
Supplement: Supplementary file 1 [file Table_1.DOCX]

***Supplementary Material***

**Altered resting-state brain activity in functional dyspepsia patients: A coordinate-based meta-analysis**

**Table S1. Quality Assessment Checklist (1 point per criterion for fully satisfied, 0.5 for partially satisfied, 0 for otherwise)**

| **Category1: Subjects**  **Score** (0/0.5/1) |
| --- |
| 1. Patients were evaluated prospectively, specific diagnostic criteria were applied, and demographic data were reported. |
| 2. Healthy subjects were evaluated prospectively, and psychiatric and medical illnesses were excluded. |
| 3. Important variables (such as age, gender, illness duration, onset time, medication status, comorbidity, and severity of illness) were checked, either by stratification or statistically. |
| 4. Sample size per group > 10. |
| **Category 2: Methods for image acquisition and analysis** |
| 5. Magnet strength ≥1.5T. |
| 6. MRI slice thickness ≤2 mm. |
| 7. The whole-brain analysis was automatically calculated with no prior regional selection.  8. Coordinates were reported in a standard space. |
| 9. The imaging technique processing was described clearly enough to be reproducible. |
| 10. Measurements were described clearly enough to be reproducible. |
| **Category 3: Results and conclusions** |
| 11. Statistical parameters were provided. |
| 12. Conclusions were consistent with the results obtained and the limitations were discussed. |
| **TOTAL**  /12 |
